# Transcriptomic Profiling of Human Peritumoral Neocortex Tissues Revealed Genes Possibly Involved in Tumor-Induced Epilepsy

**DOI:** 10.1371/journal.pone.0056077

**Published:** 2013-02-13

**Authors:** Charles E. Niesen, Jun Xu, Xuemo Fan, Xiaojin Li, Christopher J. Wheeler, Adam N. Mamelak, Charles Wang

**Affiliations:** 1 Department of Pediatrics, Cedars-Sinai Medical Center, Los Angeles, California, United States of America; 2 Medical Genetics Institute, Cedars-Sinai Medical Center, Los Angeles, California, United States of America; 3 Department of Pathology, Cedars-Sinai Medical Center, Los Angeles, California, United States of America; 4 David Geffen School of Medicine, University of California Los Angeles, Los Angeles, California, United States of America; 5 Functional Genomics Core & Department of Molecular & Cellular Biology, Beckman Research Institute, City of Hope Comprehensive Cancer Center, Duarte, California, United States of America; 6 Department of Neurosurgery, Cedars-Sinai Medical Center, Los Angeles, California, United States of America; University of Jaén, Spain

## Abstract

The molecular mechanism underlying tumor-induced epileptogenesis is poorly understood. Alterations in the peritumoral microenvironment are believed to play a significant role in inducing epileptogenesis. We hypothesize that the change of gene expression in brain peritumoral tissues may contribute to the increased neuronal excitability and epileptogenesis. To identify the genes possibly involved in tumor-induced epilepsy, a genome-wide gene expression profiling was conducted using Affymetrix HG U133 plus 2.0 arrays and RNAs derived from formalin-fixed paraffin embedded (FFPE) peritumoral cortex tissue slides from 5-seizure vs. 5-non-seizure low grade brain tumor patients. We identified many differentially expressed genes (DEGs). Seven dysregulated genes (i.e., *C1QB*, *CALCRL*, *CCR1*, *KAL1*, *SLC1A2*, *SSTR1* and *TYRO3*) were validated by qRT-PCR, which showed a high concordance. Principal Component Analysis (PCA) showed that epilepsy subjects were clustered together tightly (except one sample) and were clearly separated from the non-epilepsy subjects. Molecular functional categorization showed that significant portions of the DEGs functioned as receptor activity, molecular binding including enzyme binding and transcription factor binding. Pathway analysis showed these DEGs were mainly enriched in focal adhesion, ECM-receptor interaction, and cell adhesion molecules pathways. In conclusion, our study showed that dysregulation of gene expression in the peritumoral tissues may be one of the major mechanisms of brain tumor induced-epilepsy. However, due to the small sample size of the present study, further validation study is needed. A deeper characterization on the dysregulated genes involved in brain tumor-induced epilepsy may shed some light on the management of epilepsy due to brain tumors.

## Introduction

Epilepsy is a common complication of patients with brain tumors that causes considerable morbidity and often resists control by traditional antiepileptic drugs [1], [2]. Although it has been known for over 100 years that brain tumors cause seizures, the pathophysiology of how this occurs is still not fully understood [3]. Mass size, location, morphology and histology of tumors have been shown to be related to the frequency of epilepsy. For example, the incidence of epilepsy is much higher in low-grade gliomas than in high-grade gliomas. This may result from increased astrogliosis or partial deafferentation of circumscribed cortical tissue by slow-growing, low-grade tumors [4]–[6]. Recent studies suggest that alterations in the microenvironment of peritumoral tissue during brain tumor progression may contribute to epileptogenicity. Multiple changes have been reported in peritumoral tissues during brain tumor progression including amino acid levels, local metabolites, pH, neuronal and glial enzyme and protein expression, and density of the *N*-methyl-D-aspartate (NMDA) subclass of glutamate receptors [1], [7]. Glutamate, a major excitatory neurotransmitter, was found to be involved in brain tumor-induced peritumoral epilepsy [8], [9]. The level of glutamate was higher in the extracellular fluid overlaying epileptogenic cerebral cortex than in overlying normal tissue, whereas the intracellular to extracellular ratio of glutamine was lower in epileptiform cortex [10], [11]. In addition, both ionotropic and metabotropic glutamate receptors have been shown to be overexpressed in gliomas and peritumoral astrocytes [1], [4], [12]. Activation of glutamate receptors by glutamate could lead to down-regulation of γ-animobutyric acid (GABA)-mediated inhibitory stimuli as a second mechanism to increase seizure activity [1], [4]. A decrease in the peritumoral tissue pH, due to tumor hypoxia, can cause glial cell swelling and damage [13]. This, in turn, disrupts the distribution of sodium and calcium ions in the extracellular spaces and increases neuronal excitability [14]. Despite such biochemical and metabolic evidence, however, little is known about the alterations of gene expression and regulation in the peritumoral tissue associated with tumoral epileptogenesis.

To identify the genes potentially involved in tumor-induced epilepsy, a transcriptomic profiling using RNA derived from FFPE human peritumoral cortex tissues was conducted using Affymetrix HG U133 plus 2.0 arrays. This was the first study of such kind to use FFPE peritumoral tissues to investigate the global gene expression in brain tumor patients with epilepsy. We identified a significant number of genes differentially expressed between the seizure and non-seizure brain tumor patients. Some of the dysregulated genes could serve as good candidate for biomarkers or targets for seizure management. Our analyses also validate the notion that meaningful genomic data can be acquired from a widely available and valuable clinical resource [15]–[27].

## Results

### Quality of RNA Extracted from FFPE Tissues

**Figure 1:**
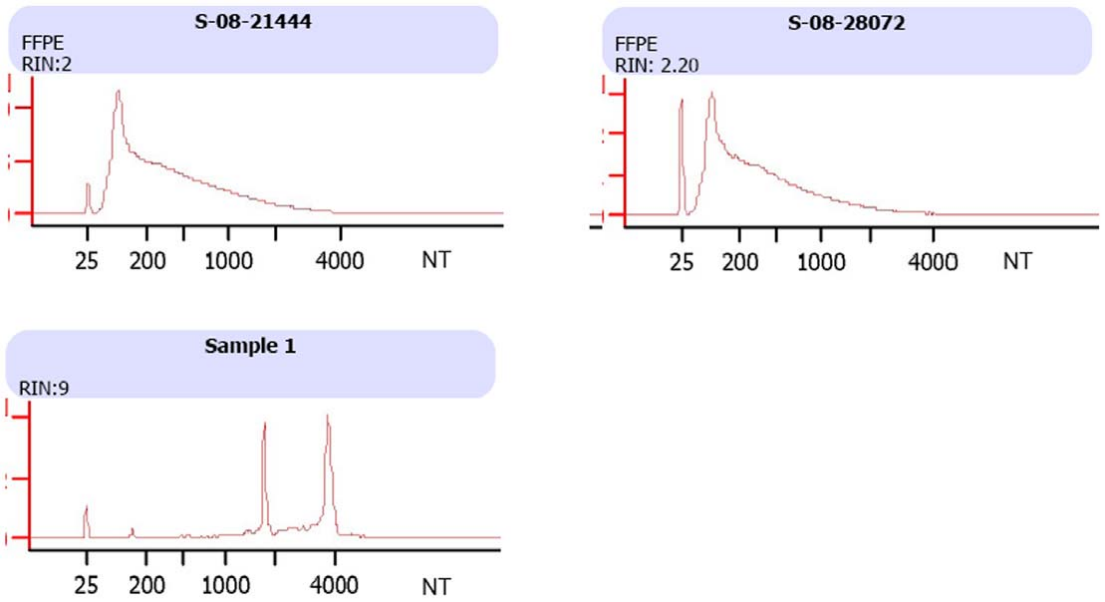
Bioanalyzer profiles of FFPE human peritumoral tissue total RNA. The total RNA was extracted from FFPE peritumoral tissues using Qiagen RNA extraction kit for FFPE samples or using Qiagen RNeasy Mini kit for human peripheral blood mononuclear cells (PBMCs). The upper panel shows the Bioanalyzer results of two FFPE RNA samples and the lower panel shows the profiling of RNA freshly extracted from human PBMCs. The Bioanalyzer profiles were obtained using Agilent RNA Nano LabChip.

Total RNA ranging from 0.67–5.74 µg (median: 2.42 µg) were obtained from 3 to 5 pieces of 10 µm thick microdissected sections of FFPE human brain peritumoral tissues using Qiagen’s RNeasy FFPE kit. RNA integrity assessed by Bioanalyzer showed that RNA extracted from FFPE tissues were significantly fragmented, with the majority of RNA sizing around 75 to 100 bp in length. The RNA integrity number (RIN), a parameter built into the Bioanalyzer software, automatically determines RNA integrity not only by the ratio of the ribosomal bands, but also by entire electrophoretic trace of the RNA sample including the presence or absence of degradation products. The RIN number of the FFPE tissues block-derived RNA ranged from 1.0 to 2.5, indicating a significant fragmentation, whereas as a control freshly isolated human peripheral blood monocyte (PBMC) RNA had excellent integrity with a RIN of 9.0 and distinctive 18s and 28s peaks (Figure 1).

### cDNA Yield and Gene Detection Rate of the FFPE Brain Tumor Tissue Block-derived RNA Samples

**Figure 2:**
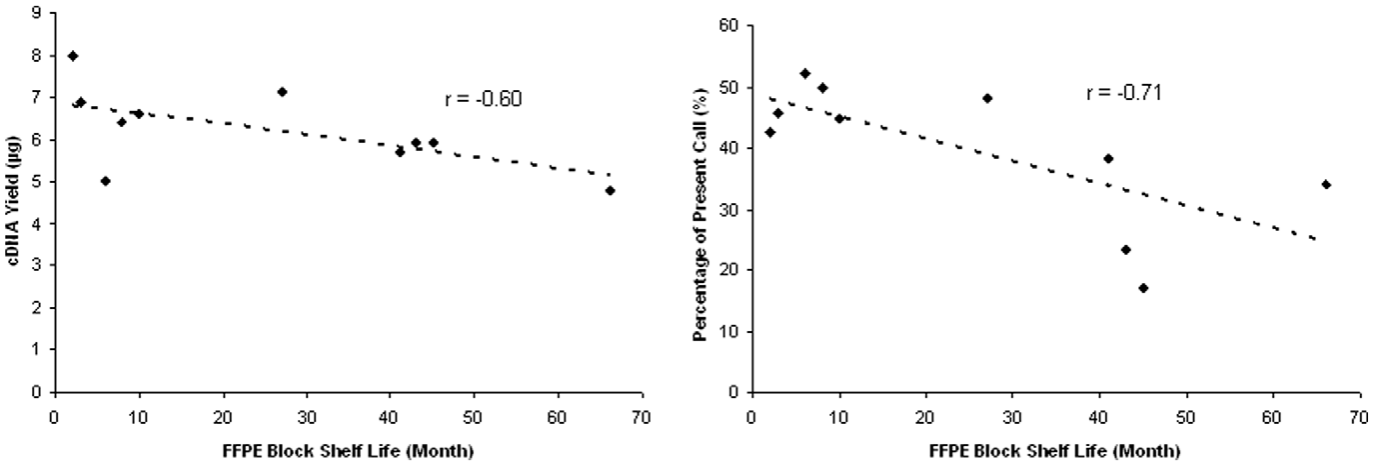
Correlations of FFPE block shelf life with cDNA yield and gene present call. Both cDNA yield and gene present call are negative correlated with FFPE block shelf life, with r = −0.60 and −0.71, respectively. Regression lines are represented as dot lines.

Table 1 shows the archive time of the FFPE human brain tissue blocks, cDNA and/or cRNA yields, 3′/5′ ratios of GAPDH and gene detection present calls of both FFPE tissue block derived RNA and PBMC RNA samples. We observed that the cDNA amplification yield from FFPE derived RNA samples was negatively correlated to the tissue block storage time (r = −0.60) with the 2-month FFPE RNA giving the highest cDNA yield (8 µg), whereas the 66-month FFPE RNA giving only 4.8 µg cDNA products despite the fact that the same amount total RNA (75 ng) was used to start the first strand cDNA synthesis. Depending on the storage time of the FFPE tissue blocks, the gene detection rates based on GCOS built-in MAS 5 algorithm showed 17.1 - 52.3% present calls in the FFPE human brain peritumoral RNA, which was also negatively correlated to the storage time of FFPE tissue blocks (r = −0.71, Figure 2). Surprisingly, the gene present calls for the most FFPE human brain peritumoral tissue samples were comparable with the freshly isolated human PBMC RNA samples (45–47%, N = 9). We speculate that although FFPE RNA appears as highly “degraded” based on Bioanalyzer QC analysis, it is most likely uniformly “fragmented” across full-length of the mRNA with the majority of the transcripts being around 75–100 bp in length and a small fraction of them retained at up to 3000–4000 bp in length. Regardless, the cDNA amplification was able to amplify both the 5′ and the 3′ regions of the transcripts with similar efficiency, as an excellent 3′/5′ ratio of housekeeping gene GAPDH was also obtained (0.6–3.4) from Affymetrix GeneChips. There was no significant difference in FFPE block storage time between the epilepsy and non-epilepsy groups (*p*>0.05, Student *t*-test).

**Table 1:** cDNA yield and gene detection rate of FFPE brain peritumoral tissues derived RNA samples.

Sample§	Tissue Storage Time (Month)	RNA		cDNA or cRNA			Present Call (%)
		OD260/280	Input (ng)	OD260/280	Yield (µg)	GAPDH 3'/5' Ratio	
Epil	8	2.1	75	2.0	6.4	1.8	49.8
Epil	41	2.2	75	2.0	5.7	1.8	38.2
Epil	66	2.1	75	2.1	4.8	3.4	34.1
Epil	2	2.1	75	2.1	8.0	1.4	42.5
Epil	27	2.2	75	2.1	7.1	1.5	48.1
Nonepil	6	2.2	75	2.1	5.0	1.8	52.3
Nonepil	43	2.1	75	2.1	5.9	0.6	23.5
Nonepil	45	2.0	75	2.1	5.9	1.6	17.1
Nonepil	3	2.0	75	2.0	6.9	2.7	45.8
Nonepil	10	2.0	75	2.0	6.6	2.3	44.6
PBMC*	fresh	2.1	300	2.0–2.2	80–100¥	1.7–2.0	45–47

§ Sample: Epil, samples from patient with epilepsy; Nonepil, samples from patient without epilepsy.

* PBMC, fresh human PBMC samples from 9 different patients.

¥ cRNA.

### Differentially Expressed Genes (DEGs) and the Dysregulated Genes Associated with Epilepsy Supported by Literature

We used both the parametric no paired Student T-test (2-fold plus p≤0.05, no FDR applied) [28], [29] and the non-parametric Rank Product, i.e., the Bioconductor RankProd package with a FDR ≤0.3 [30], to identify the DEGs between the two groups. We identified 345 probe sets (representing 296 genes) using the Student T-test (Table S1) as compared with 377 probe sets (representing 344 genes) from the RankProd (Table S2). The Bioconducdtor RankProd package identified DEGs were listed in the Table S2 and the P values produced from the Bioconductor RankProd were also included in the Table 2 along with the P values from T-test. There was 41% overlapping between the T-test (FC plus P value) and RankProd DEG lists, which somehow validated the DEGs produced from the Student T-test. This also suggests that the FC (≥2) plus P value (≤0.05, Student T-test) criteria is somehow more stringent than RankProd when applied to our human microarray data as no DEGs would come out if applied to a FDR using the Student T-test. We further did a thorough literature review and compared the significantly differentially expressed genes list (DEGs) identified by our microarray study with what were reported in the literature. The Table 2 summarized some dysregulated genes and their roles or associations with epilepsy including the fold changes, P values along with references which were also listed in the bibliography. Some dysregulated genes were further discussed in our discussion.

**Table 2:** Dysregulated genes associated with epilepsy documented in literature.

Gene	FC*	P*	P§	Role or association with epilepsy	References¥
ADRA1A	2.5	0.024	N/A	Involves in seizure-associated process	Gundlach AL, et al. (1995), Brain Res 672: 214–227 [31]
					Blendy JA, et al. (1990), J Neurosci 10: 2580–2586 [32]
				Inhibits seizure responses in animal models of epilepsy	Rutecki PA, et al. (1995), Epilepsy Res 20: 125–136 [33]
GALR1	−2	0.034	N/A		Lerner JT, et al. (2008), Cell Mol Life Sci 65: 1864–1871 [34]
					Sadegh M, et al. (2007), Neuroscience 150: 396–403 [35]
ARHGAP18	−3	0.004	0.00004	Regulates neuronal excitability	Mathie A (2007), J Physiol 578: 377–385 [36]
ARHGEF12	2.1	0.011	N/A		
CAMK1	2.6	0.028	N/A	Changes neuronal excitability and the frank epileptiform discharges	Churn SB, et al. (2000), PNAS USA 97: 5604–5609 [37], [38]
CCR1	−3.3	0	2.71E-05	Regulates neuronal excitability	Mathie A (2007), J Physiol 578: 377–385 [36]
P2RY12	2	0.028	N/A		
RGS7	2.2	0.048	N/A		
KAL1	3	0.025	0.0002	Regulates neuronal migration	del Castillo I, et al. (1992), Nat Genet 2: 305–310 [39]
				Regulates fibroblast growth factor signaling pathway activation	Hu Y, et al. (2009), J Biol Chem 284: 29905–29920 [40]
					Jian B, et al. (2009), Cell Cycle 8: 3770–3776 [41]
				Increases neuron excitability and seizure	Zucchini S (2008), J Neurosci 28: 13112–13124 [42]
NAPA	−2.2	0.024	0.0002	Ceases neurotransmitter release	Matveeva EA, et al. (2007), Epilepsy Res 73: 266–274 [43]
					Whiteheart SW, et al. (2001), Int Rev Cytol 207: 71–112 [44]
SLITRK2	2.2	0.034	N/A	Affects activity transduction	Aruga J, et al. (2003), Mol Cell Neurosci 24: 117–129 [45]
ITGB1	−2.4	0.022	0.0014	Stabilizes activity-induced increases in synaptic strength and excitability	Gall CM, et al. (2004), Adv Exp Med Biol 548: 12–33 [46]
ITGB2	−2.1	0.012	N/A		
CDH11	−3	0.005	0.0005		
NID1	−2.8	0.008	0.0003	Inhibits epileptic activity	Kohling R, et al. (2006), Neurodegener Dis 3: 56–61 [47]
PCDH15	2.8	0.008	0.0003	Regulates blood brain barrier integrity	Librizzi L, et al. (2006), Epilepsia 48: 743–751 [48]
PCDHB9	2.4	0.036	N/A		Dietrich JB (2002), J Neuroimmunol 128: 58–68 [49]
CADM1	2	0.02	N/A		
C1QA	−2.1	0.038	N/A	Eliminates synapses and allow maturepatterns of neuronal connectivity	Huh GS, et al. (2000), Science 290: 2155–2159 [50]
C1QB	−3.1	0.016	0.0003		Stevens B, et al. (2007), Cell 131: 1164–1178 [51]
C1S	−3.7	0.016	3.00E-05	Promotes epileptic seizure	Chu Y, et al. (2010), PNAS USA 107: 7975–7980 [52]
CFH	−3.9	0.03	1.72E-05		Salin P, et al (1995), J Neurosci 15: 8234–8245 [53]
CFH/CFHR1	−4	0.028	N/A		Prince DA, et al (1993), J Neurophysiol 69: 1276–1291 [54]
CDH11	−2.8	0.005	0.0005	Modulates synaptic plasticity, neuronalexcitability and homeostasis	Dityatev A (2010), Epilepsia 51 Suppl 3: 61–65 [Ref.: 55]
AMIGO2	−2.5	0.033	0		Dityatev A (2006), Results Probl Cell Differ 43: 69–97 [56]
ATP7A	−2.3	0.043	0.0012	Associated with hyperexcitability in epilepsy	Heck N, et al (2004), Neuroscience 129: 309–324 [57]; Elmer E, et al (1997), Neuroreport 8: 1193–1196 [58]; Veznedaroglu E (2002), J Neurosurg 97: 1125–1130 [59]
CD93	−2.9	0.002	0.0002		
CLDN23	−2.4	0.035	0.0004		
COL1A1	−4	0.03	0.002		
COL1A2	−4	0.039	0.001		
COL3A1	−8.6	0.023	0.001		
COL5A2	−2.6	0.02	0.001		
DCN	−5.4	0.025	0.001		
ITGB1	−2.4	0.022	0.0014		

* FC ≥2 plus P≤0.05, unpaired Student T-test.

§ Non-parametric RankProd test and corresponding P value. N/A, not available and not identified by the RankProd approach.

¥ The number in the square brackets refers to the citation number in the bibliography in the paper.

### Principle Component Analysis (PCA)

**Figure 3:**
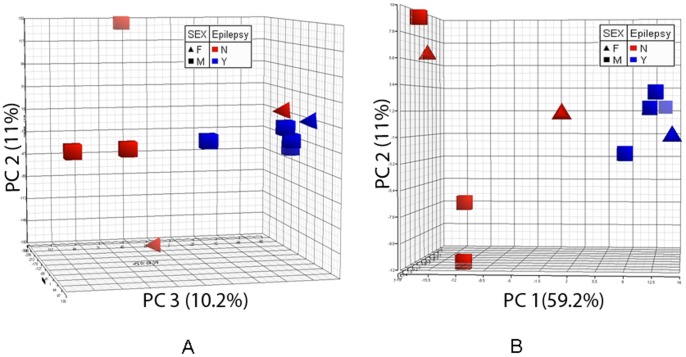
3-D View of Principal Component Analysis showing distinguished clusters between epilepsy and non-epilepsy subjects. The PCA was performed based on all genes (A) and differentially expressed genes (B, DEGs, ≥2-fold change plus p≤0.05, T-test) between epilepsy and non-epilepsy patients. Patients with or without epilepsy are represented by different colors, i.e., blue for epilepsy (Y); red for non-epilepsy (N). The gender is represented by different symbols, i.e., square for male (M); triangle for female (F).

PCA is a mathematical technique to project the observations (samples) from the high-dimensional variables (genes) space to a low-dimensional subspace spanned by several linear combinations of the original variables (genes) to account for the maximum variability in the data sets [60], [61]. PCA has been widely used to analyze and visualize multidimensional data sets [62], [63]. We first performed a PCA using all genes to visualize the global distribution of various FFPE samples. As shown in Figure 3A, 4 out of 5 non-epilepsy subjects formed a distinct cluster away from the epilepsy subjects at PC2 and PC3 panel, which accounted for 11% and 10.2% of total variance, respectively. Epilepsy subjects clustered more closely to each other than non-epilepsy subjects. This distribution pattern suggested that a significant difference in gene expressions exists between the two groups. In addition, PCA performed on the DEGs showed that the epilepsy subjects were separated from the non-epilepsy subjects, forming two distinguishable clusters along PC1 axis in PC1 and PC2 panel (Figure 3B), which accounted for the majority of variation (59.2%). Furthermore, the samples from the epilepsy group were again clustered more closely as compared with the non-epilepsy, indicating the consistency of the DEGs identified by our gene expression profiling.

### Microarray Gene Expression Validation by Real-time qRT-PCR

Seven DEGs, i.e., *C1QB*, *CALCRL*, *CCR1*, *KAL1*, *SLC1A2*, *SSTR1*, *TYRO3*, identified by microarray were further subject to validation by real-time qRT-PCR using RNA extracted from microdissected tissue sections from the same FFPE tissue blocks. The selection of the genes for qRT-PCR validation were based on (1) significant fold change (FC) in the microarray, i.e., FC ≥ ±2 plus P≤0.05; and/or (2) the molecular functions of the genes related to seizure including evidence from the previous reported studies, i.e., C1QB, CCR1 and KAL1. Comparison of the fold changes including the means, standard deviations (SD) and P values between the microarray and qRT-PCR was shown in Table 3. We observed a remarkably high concordance in the FCs between microarray and qRT-PCR for both down-regulated and up-regulated genes examined (R = 0.918, p = 0.0085, Person’s correlation).

**Table 3:** Microarray identified differentially expressed genes validated by real-time qRT-PCR.

Gene	Molecular Function Involved	Microarray^#^			qRT-PCR^#^	
		Fold Change*(Mean ± SD)	P*	P^§^	Fold Change (Mean ± SD)	P*
C1QB	Eliminates synapses and allows mature patterns of neuronal connectivity	−3.1±0.81	0.016	0.0003	−2.2±1.07	0.019
CALCRL	Involved in generation of cAMP	−2.9±0.78	0.020	0.0005	−1.6±0.85	0.278
CCR1	Regulates neuronal excitability	−3.2±0.85	0.000	2.7E-5	−2.0±1.08	0.006
KAL1	Increases neuron excitability and seizure	3.0±0.79	0.025	0.0002	2.1±1.73	0.011
SLC1A2	Clears glutamate from the extracellular space at synapses	2.0±0.43	0.129	N/A	5.0±1.51	0.010
SSTR1	Inhibits the release of many hormones and other secretory proteins	2.7±0.72	0.034	N/A	4.3±1.38	0.002
TYRO3	Transduces signals from the extracellular matrix into the cytoplasm	2.3±0.51	0.033	N/A	2.3±1.07	0.044

The real-time RT-PCR was performed using QuantiFast Probe Assays (Qiagen, Valencia, CA) and the housekeeping gene *GAPDH* was used as endogenous control. SD: standard deviation. ^#^Person’s correlation was carried using the mean fold changes between microarray and qRT-PCR with an R = 0.918, p = 0.0085. *Unpaired Student T-test was applied. **^§^**Non-parametric RankProd package test and P value. N/A, not available.

### Gene Ontology and Pathway Analysis

Using DAVID Bioinformatics Resources [64], [65], DEGs were further dissected based upon gene ontology classification and biological pathways against several public databases with default EASE score, which is a modified Fisher Exact probability p-value for gene-enrichment analysis. The results of biological processes and molecular function of the DEGs and related signaling pathways derived from DAVID Bioinformatics Resources (data not shown) indicated that a considerable numbers of genes were categorized as signal transduction (*p* = 0.005), cell differentiation (*p* = 0.004), regulation of immune system process (*p* = 0.005), cell-cell adhesion (*p* = 0.002), extracellular matrix organization (*p* = 1×10^−5^). Molecular functional categorization (data not shown) showed that significant portions of the DEGs were grouped as receptor activity (*p* = 0.04), molecular binding including enzyme binding (*p* = 0.002), transcription factor binding (*p* = 0.003), growth factor binding (*p* = 0.0004), SMAD binding (*p* = 0.004) and transcription coactivator activity (*p* = 0.003). Pathway analysis against KEGG database showed these DEGs were mainly enriched in focal adhesion (*p* = 0.001), ECM-receptor interaction (*p* = 2.7×10^−5^), and cell adhesion molecules (*p* = 0.003) pathways. The functional and pathway analysis results from DAVID Bioinformatics Resources are consistent with the previous finding that disordered neuronal connectivity and regulation, impaired glial cell function, and the presence of altered vascular supply and permeability may contribute to peritumoral epilepsy [1].

## Discussion

Epilepsy associated with brain tumor carries greater morbidity [1]. Although the mechanism is yet unclear; it has been shown that location, histology and size of the tumor as well as peritumoral environment changes may contribute to epileptogenesis [1]. Transcriptomic study allows investigation simultaneously of thousands of genes, providing a powerful tool to explore the molecular mechanism of brain tumor-induced epilepsy. Currently, however, many transcriptomic studies rely on high integrity of the RNA samples, limiting the use of clinical samples, particularly from FFPE tissue blocks in which transcripts may be “degraded” due to formalin cross-linking and chemical modification [17]–[19]. Recent studies demonstrated the feasibility of profiling FFPE vs. fresh frozen tissue RNA by microarray [16]–[17], [24]–[26]. Here we used RNA derived from FFPE human brain tumor tissue blocks to study the molecular mechanism of brain tumor related epilepsy. Similar to previous reports [66], [67], RNA extracted from FFPE sections are smaller and lack 18 s and 28 s ribosomal RNA peaks on Bioanalyzer profiling, compared to RNA isolated from fresh viable PBMC (Figure 1). It has been reported that the microarray probe performance of FFPE RNA profiling was more significantly affected by the proximity of the probes to the 3′ end of RNA transcripts and declined sharply toward 5′ end, suggesting the FFPE preservation induced 5′ to 3′ chemical degradation to RNA from chemical fixation and paraffin embedding [15]. However, this was not observed in our study. The ratio of 3′/5′ signal intensity of housekeeping gene GAPDH ranged from 0.6 to 3.4, which is comparable to that from fresh frozen samples. This finding was further supported by real-time PCR using primers targeting both 3′ and 5′ ends of another housekeeping gene *actin* (data not shown). Furthermore, our qRT-PCR validation on seven DEGs from microarray using the RNA extracted from the same FFPE tissue blocks showed an excellent concordance in the FCs between the microarray and qRT-PCR. In addition, the global gene expression profiling of the peritumoral tissues from brain tumor patients with epilepsy showed distinct clusters in PCA analysis, compared to that from patients without epilepsy episodes, suggesting there is a substantial transcriptional alteration in the potentially epileptogenic peritumoral tissues (Figure 3). Using both the parametric Student T-test (2-fold plus p≤0.05) [28], [29] and the non-parametric Rank Product, i.e., the Bioconductor RankProd package [30], we identified many DEGs between the two groups (RankProd, 345; T-test, 296). The most significant functional groups were discussed briefly below, and listed in Table 2, with a detailed list of all DEGs provided in supplemental Tables. We would like to acknowledge that the sample size of our study was small because we used high stringent criteria in selecting subjects to control confounding factors. Thus, the effects due to the brain tumor type and location could not be evaluated.

Several of the dysregulated genes identified by our microarray study have been previously implicated in epileptic seizure or related activities, serving to further validate our analysis. For example, electroconvulsive shock and stimulation evoked seizures produced significant increase in adrenergic receptor *alpha*-1 densities including *alpha* 1a receptor [31], [32], which was up-regulated 2.5-fold in peritumoral tissues in our study. Moreover, *ADRA1A* and Galanin were shown to inhibit seizure responses in animal models of epilepsy [33]–[35]. The cell membrane protein genes along with some other differentially expressed genes including Rho quinine nucleotide exchange factor (*ARHGEF12*, 2.1-fold), Rho GTPase activating protein 18 (*ARHGAP18*, −3.0-fold), chemokine (C-C motif) receptor 1 (*CCR1*, −3.2-fold), purinergic receptor P2Y (*P2RY12*, 2.0-fold) and regulator of G-protein signaling 7 (*RGS7*, 2.2-fold) are known to coordinate signaling through G-protein coupled receptor protein signaling pathway that leads to cellular ion channel regulation [36], hence neuronal excitability regulation. In addition, increased expression of calcium/calmodulin-dependent protein kinase 1 (*CAMK1*, 2.6-fold) and calcium/calmodulin-dependent protein kinase II inhibitor 1 (*CAMK2N1*, 2.1-fold) that results in alteration of calcium homeostasis may also contribute to the change of neuronal excitability and the frank epileptiform discharges [37], [38] associated with brain tumors.

Down-regulation of adhesion molecules, including integrin (*ITGB1, ITGB2*) and cadherin family members (*CDH11*) in the peritumoral tissue of the subjects with epilepsy also serve to validate our study, as they have also been found to contribute to the cell biology underlying epileptogeneis [46]. In contrast, up-regulation of cell adhesion molecule 1 (*CADM1*, 2.0-fold), protocadherin 15 (*PCDH15*, 2.8-fold) and protocadherin beta 9 (*PCDHB9*, 2.4-fold) may be relevant because epileptiform activity is known to induce the expression of adhesion molecules [48], some of which regulate blood brain barrier integrity [49]. Dysregulation of some adhesion molecules in the peritumoral tissue may also contribute to the epileptic episodes in these patients.

Many other dysregulated genes identified by our microarray study and their possible involvements in the brain tumor induced epilepsy were summarized in the Table 2, not discussed here. The Table 2 also included the publications supporting the role of these genes involved in epilepsy and the referred papers were listed in the reference.

In conclusion, our present study identified many dysregulated genes and signaling cascades in the human brain peritumoral tissues. This extends our knowledge on genes previously implicated in the pathogenesis of the human brain tumor-induced seizures and may shed some light on the management of epilepsy due to brain tumors. We acknowledge the limitation of our small sample size due to the high stringency of selecting study patients, thus further biological validation is needed. Nevertheless, this was the first study of such kind to use FFPE peritumoral tissues to investigate the global gene expression in brain tumor patients with epilepsy to identify dysregulated genes related to epilepsy.

## Materials and Methods

### Subjects

Archived FFPE tissue blocks from 10 patients who underwent brain surgery at Cedars-Sinai Medical Center, Los Angeles, CA, for various low grade brain tumors during the period from 2003 to 2008 were selected according to patient’s history of epilepsy. Patients diagnosed with primary brain tumors who received preoperative neoadjuvant chemotherapy or radiation therapy were excluded from the present study. The patients with metastatic brain tumors were also excluded. In addition, in the epilepsy group, we only included the patients with a seizure onset in the early stage of seizure attacks. Patient’s characteristics including the age, sex, type and location of brain tumors and the status of epilepsy are listed in Table 4. As shown in the Table 4, five patients had documented history of epileptic episodes; the other 5 patients had no known history of epilepsy. Patients in the epilepsy group were selected based on early surgery treatment and low seizure frequency. Three of the five patients had surgery less than one year after seizure presentation. Four of the five epilepsy patients averaged less than one seizure per month till surgery. The fifth patient had recurrent seizures on a biweekly basis for over two years. All tissue blocks were carefully investigated by an experienced neruopathologist to locate the brain peritumoral tissues and 3 to 5 pieces of 10 µm sections of the peritumoral tissues were microdissected and transferred immediately into RNase free microcentrifuge tubes (USA Scientific, Orlando, FL). The tubes were sealed immediately to prevent excess air exposure and transferred to the lab for RNA extraction.

**Table 4:** Clinical characteristics of study subjects.

Epilepsy	Age (year)	Gender	Tumor Site	Tumor Type
Y	7.7	F	L. T	OLG
Y	8.9	M	R. T	AST
Y	34.5	M	L. T	AST-gr 2
Y	6.5	M	L. T	GGL
Y	6.5	M	L. Oc	GGL
N	13.5	F	R. Fr	OLG
N	18.1	F	L. FP	GGL
N	33.2	M	L. Fr	GGL
N	26.5	M	L. PT	GGL
N	15	M	R. T	AST-gr 2

Epilepsy status: Y, brain tumor patient with epilepsy; N, brain tumor patient with no epilepsy. Tumor site: L, left; R: right; T, temporal lobe; Oc, occipital lobe; Fr, frontal lobe; FP, frontal parietal lobe; PT, parietal temporal lobe. Tumor type: OLG, oligodendroglioma; AST-gr 2, astrocytoma grade 2; GGL, ganglioglioma.

### Ethics Statement

The study design and protocols of patient sample selection, sample processing and experimental procedures were approved by Institutional Review Board at Cedars-Sinai Medical Center. Written or verbal consent was not obtained for two reasons. First, this was a retrospective study in which only the archived FFPE samples were used and there was no way to contact patients and there was no impact on the treatment. Second, the patients’ identify information was de-identified by a special code so that other research staff carrying out RNA extraction and gene expression had no access to the patients’ information. The Institutional Review Board at Cedars-Sinai Medical Center waived the need for consent for the reasons mentioned above. All procedure was carefully reviewed and approved by Institutional Review Board at Cedars-Sinai Medical Center.

### RNA Extraction from FFPE Samples

RNA was extracted from 3 to 5 microdissected sections of FFPE human brain peritumoral tissue blocks using RNeasy FFPE Kit (Qiagen, Valencia, CA) following manufacturer’s instructions. Briefly, tissue sections were mixed with 1 ml xylene to remove paraffin by vortexing for 10 seconds followed by centrifugation for 2 minutes. Residual xylene in the resulting tissue pellets was removed with 100% ethanol. Tissue pellets were then incubated with proteinase K at 55°C for 15 minutes followed by 80°C for 15 minutes to reverse formaldehyde modification of nucleic acids. After incubation, tissues were lysed with buffer solution and genomic DNA was removed by passing the lysates through gDNA eliminator spin column coming with the RNeasy FFPE kit. The flow-through was mixed with 100% ethanol and loaded on RNeasy MinElute spin column to capture the RNA. After centrifugation, the spin column with RNA binding on it was washed with buffer RPE and RNA was eluted with RNase-free water. Quantities and purity of RNA were checked with spectrometer. Agilent 2100 Bioanalyzer (Agilent Technologies, Wilmington, DE) was used to check RNA integrity before cDNA synthesis. An additional RNA sample extracted from freshly isolated human peripheral blood mononuclear cells (PBMC) using Qiagen RNeasy mini kit was run in parallel with RNA extracted from FFPE tissues as normal controls.

### cDNA Synthesis, Amplification and Labeling

cDNA was amplified and biotin labeled using WT-Ovation FFPE RNA amplification system and FL-Ovation cDNA biotin module (NuGen, San Carlos, CA), respectively. Seventy-five nanograms total RNA was used to generate first strand cDNA with 2.5 µl first strand buffer mix and 0.5 µl first strand enzyme mix incubated at 4°C for 2 minutes, 25°C for 30 minutes, 42°C for 15 minutes and 70°C for 15 minutes. Then 9.75 µl of second strand buffer mix and 0.25 µl second strand enzyme mix were added into the reaction and incubated at 4°C for 1 minute, 25°C for 10 minutes, 50°C for 30 minutes and 70°C for 5 minutes to synthesize the second strand cDNA. Double strand cDNA was purified using Agencourt RNAClean beads according to manufacture’s protocol. cDNA amplification was performed first by mixing purified cDNA with 30 µl SPIA buffer mix, 20 µl SPIA primer mix 1, 0.7 µl SPIA enhancer and 10 µl SPIA enzyme mix and incubated at 4°C for 1 minute followed by 47°C for 30 minutes. Then 30 µl more SPIA buffer mix was added into the reaction along with 20 µl SPIA primer mix 2, 2.3 µl SPIA enhancer, 30 µl SPIA enzyme mix and incubated at 4°C for 1 minute, 47°C for 60 minutes and 95°C for 5 minutes. Amplified cDNA was purified with RNeasy MinElute cleanup columns (Qiagen, Valencia, CA) according to manufacture’s protocol. Five micrograms purified cDNA was fragmented in 5 µl fragmentation buffer mix plus 2 µl fragmentation enzyme mix and incubated at 37°C for 30 minutes and 95°C for 2 minutes. Biotin labeling was performed by mixing fragmented cDNA with 15 µl labeling buffer mix, 1.5 µl labeling reagent, 1.5 µl labeling enzyme mix and incubated at 37°C for 60 minutes and 70°C for 10 minutes.

### DNA Microarray and Data Analysis

The Affymetrix Human Genome U133 plus 2.0 GeneChips were used for expression profiling according to the procedure as described previously [68]. Five micrograms of biotinylated fragmented cDNA were hybridized with the Genechips. After washing, the arrays were stained with streptavidin-phycoerythrin (Molecular Probes, Eugen, OR). Signals were amplified by biotinylated anti-streptavidin (Vector Laboratories, Inc., Burlingame, CA), and then scanned on Affymetrix GeneChip Scanner 3000 7G controlled by Affymetrix GCOS software. Default probe level summarization algorithm used in GCOS is MAS5, which gives gene detection calls (present, absent or marginal) to each probe set in addition to signal intensity. All data is MIAME compliant and the raw data has been deposited in a MIAME compliant database, i.e., GEO (access #: GSE32534).

We used Partek Genomics Suite (St. Louis, Missouri) and the Bioconductor RankProd package [30] for microarray data analysis. Robust Multiarray Averaging (RMA) algorithm was used for probe level summarization and cross-array normalization. Principle Component Analysis was used for visualization of sample distribution. The criteria of selecting DEGs was preset as either 2-fold plus p≤0.05, unpaired Student T-test (no FDR applied) or FDR ≤0.3, non-parametric Rank Product approach, the Bioconductor RankProd package [30], between two groups. DEGs were submitted to David Bioinformatics Resources 6.7, an online bioinformatics database for gene ontology and pathway analysis using default settings [64], [65].

### Real-time RT-PCR Validation of Selected DEGs

To validate the microarray gene expression data, seven DEGs identified by microarray were validated by real-time RT-PCR using QuantiFast Probe Assays (Qiagen, Valencia, CA). RT- PCR was performed in a one-step RT-PCR process according to the protocol on ABI 7900HT (Applied Biosystems, Foster City, CA) using 30 ng RNA extracted from the microdissected peritumoral tissue sections of the same tissue blocks stored for more than additional 3 years since the microarrays were carried out. Housekeeping gene *GAPDH* was used as endogenous control. RNA was first reverse transcribed into cDNA at 50°C for 20 min. After enzyme activation at 95°C for 5 min, PCR was carried out at 95°C for 15 s and 60°C for 30 s for 40 cycles. Comparative Ct method (delta delta Ct method) was used to calculate the fold differences between epilepsy and non-epilepsy groups.

## Supporting Information

Table S1 **Differentially expressed genes of peirtumoral tissues between epilepsy and non-epilepsy.** *Student T-test, (no FDR applies), FC ≥ ±2 plus P≤0.05.
(XLSX)

Table S2 **RankProd identified DEGs of peirtumoral tissues between epilepsy and non-epilepsy.** *Non-parametric Rank Product approach, the Boconductor RankProd with a FDR = 0.3 applied to identify the DEGs.
(XLSX)

## References

[1] Shamji MF, Fric-Shamji EC, Benoit BG (2009) Brain tumors and epilepsy: pathophysiology of peritumoral changes. Neurosurg Rev 32: 275–284; discussion 284–286. 10.1007/s10143-009-0191-7 19205766

[2] Kurzwelly D, Herrlinger U, Simon M (2010) Seizures in patients with low-grade gliomas–incidence, pathogenesis, surgical management, and pharmacotherapy. Adv Tech Stand Neurosurg 35: 81–111. 20102112 10.1007/978-3-211-99481-8_4

[3] Rajneesh KF, Binder DK (2009) Tumor-associated epilepsy. Neurosurg Focus 27: E4. 10.3171/2009.5.FOCUS09101 19645560

[4] Ruda R, Trevisan E, Soffietti R (2010) Epilepsy and brain tumors. Curr Opin Oncol 22: 611–620. 20706121 10.1097/CCO.0b013e32833de99d

[5] van Breemen MS, Wilms EB, Vecht CJ (2007) Epilepsy in patients with brain tumours: epidemiology, mechanisms, and management. Lancet Neurol 6: 421–430. 17434097 10.1016/S1474-4422(07)70103-5

[6] Wolf HK, Roos D, Blumcke I, Pietsch T, Wiestler OD (1996) Perilesional neurochemical changes in focal epilepsies. Acta Neuropathol 91: 376–384. 8928614 10.1007/s004010050439

[7] Beaumont A, Whittle IR (2000) The pathogenesis of tumour associated epilepsy. Acta Neurochir (Wien) 142: 1–15. 10664370 10.1007/s007010050001

[8] Buckingham SC, Campbell SL, Haas BR, Montana V, Robel S, et al (2011) Glutamate release by primary brain tumors induces epileptic activity. Nat Med 17: 1269–1274. 21909104 10.1038/nm.2453 PMC3192231

[9] deGroot J, Sontheimer H (2011) Glutamate and the biology of gliomas. Glia 59: 1181–1189. 21192095 10.1002/glia.21113 PMC3107875

[10] Hamberger A, Nystrom B, Larsson S, Silfvenius H, Nordborg C (1991) Amino acids in the neuronal microenvironment of focal human epileptic lesions. Epilepsy Res 9: 32–43. 1909237 10.1016/0920-1211(91)90044-g

[11] Ricci R, Bacci A, Tugnoli V, Battaglia S, Maffei M, et al (2007) Metabolic findings on 3T 1H-MR spectroscopy in peritumoral brain edema. AJNR Am J Neuroradiol 28: 1287–1291. 17698529 10.3174/ajnr.A0564 PMC7977674

[12] Aronica E, Yankaya B, Jansen GH, Leenstra S, van Veelen CW, et al (2001) Ionotropic and metabotropic glutamate receptor protein expression in glioneuronal tumours from patients with intractable epilepsy. Neuropathol Appl Neurobiol 27: 223–237. 11489142 10.1046/j.0305-1846.2001.00314.x

[13] Kempski O, Staub F, Jansen M, Schodel F, Baethmann A (1988) Glial swelling during extracellular acidosis in vitro. Stroke 19: 385–392. 3354026 10.1161/01.str.19.3.385

[14] Hossmann KA, Seo K, Szymas J, Wechsler W (1990) Quantitative analysis of experimental peritumoral edema in cats. Adv Neurol 52: 449–458. 2396538

[15] Abdueva D, Wing M, Schaub B, Triche T, Davicioni E (2010) Quantitative expression profiling in formalin-fixed paraffin-embedded samples by affymetrix microarrays. J Mol Diagn 12: 409–417. 20522636 10.2353/jmoldx.2010.090155 PMC2893624

[16] Frank M, Doring C, Metzler D, Eckerle S, Hansmann ML (2007) Global gene expression profiling of formalin-fixed paraffin-embedded tumor samples: a comparison to snap-frozen material using oligonucleotide microarrays. Virchows Arch 450: 699–711. 17479285 10.1007/s00428-007-0412-9

[17] Scicchitano MS, Dalmas DA, Bertiaux MA, Anderson SM, Turner LR, et al (2006) Preliminary comparison of quantity, quality, and microarray performance of RNA extracted from formalin-fixed, paraffin-embedded, and unfixed frozen tissue samples. J Histochem Cytochem 54: 1229–1237. 16864893 10.1369/jhc.6A6999.2006

[18] Werner M, Chott A, Fabiano A, Battifora H (2000) Effect of formalin tissue fixation and processing on immunohistochemistry. Am J Surg Pathol 24: 1016–1019. 10895825 10.1097/00000478-200007000-00014

[19] Fowler CB, O’Leary TJ, Mason JT (2008) Modeling formalin fixation and histological processing with ribonuclease A: effects of ethanol dehydration on reversal of formaldehyde cross-links. Lab Invest 88: 785–791. 18490897 10.1038/labinvest.2008.43

[20] Stanta G, Schneider C (1991) RNA extracted from paraffin-embedded human tissues is amenable to analysis by PCR amplification. Biotechniques 11: 304, 306, 308. 1718327

[21] Krafft AE, Duncan BW, Bijwaard KE, Taubenberger JK, Lichy JH (1997) Optimization of the isolation and amplification of RNA from formalin-fixed, paraffin-embedded tissue: The Armed Forces Institute of Pathology Experience and Literature Review. Mol Diagn 2: 217–230. 10462613 10.1054/MODI00200217

[22] Lee CH, Bang SH, Lee SK, Song KY, Lee IC (2005) Gene expression profiling reveals sequential changes in gastric tubular adenoma and carcinoma in situ. World J Gastroenterol 11: 1937–1945. 15800983 10.3748/wjg.v11.i13.1937 PMC4305714

[23] Onken MD, Worley LA, Ehlers JP, Harbour JW (2004) Gene expression profiling in uveal melanoma reveals two molecular classes and predicts metastatic death. Cancer Res 64: 7205–7209. 15492234 10.1158/0008-5472.CAN-04-1750 PMC5407684

[24] Mittempergher L, de Ronde JJ, Nieuwland M, Kerkhoven RM, Simon I, et al (2011) Gene expression profiles from formalin fixed paraffin embedded breast cancer tissue are largely comparable to fresh frozen matched tissue. PLoS One 6: e17163. 21347257 10.1371/journal.pone.0017163 PMC3037966

[25] Budczies J, Weichert W, Noske A, Muller BM, Weller C, et al (2011) Genome-wide gene expression profiling of formalin-fixed paraffin-embedded breast cancer core biopsies using microarrays. J Histochem Cytochem 59: 146–157. 21339180 10.1369/jhc.2010.956607 PMC3201135

[26] Berg D, Wolff C, Malinowsky K, Tran K, Walch A, et al (2012) Profiling signalling pathways in formalin-fixed and paraffin-embedded breast cancer tissues reveals cross-talk between EGFR, HER2, HER3 and uPAR. J Cell Physiol 227: 204–212. 21391216 10.1002/jcp.22718

[27] Jacobson TA, Lundahl J, Mellstedt H, Moshfegh A (2011) Gene expression analysis using long-term preserved formalin-fixed and paraffin-embedded tissue of non-small cell lung cancer. Int J Oncol 38: 1075–1081. 21305253 10.3892/ijo.2011.936

[28] Guo L, Lobenhofer EK, Wang C, Shippy R, Harris SC, et al (2006) Rat toxicogenomic study reveals analytical consistency across microarray platforms. Nat. Biotechnol. 24: 1162–1169. 10.1038/nbt1238 17061323

[29] MAQC Consortium, Shi L, Reid LH, Jones WD, Shippy R, et al (2006) The MicroArray Quality Control (MAQC) project shows inter- and intraplatform reproducibility of gene expression measurements. Nat. Biotechnol. 24: 1151–1161. 10.1038/nbt1239 PMC3272078 16964229

[30] Hong F, Breitling R, McEntee CW, Wittner BS, Nemhause JL, et al (2006) RankProd: a bioconductor package for detecting differentially expressed genes in meta-analysis. Bioinformatics 22: 2825–2827. 16982708 10.1093/bioinformatics/btl476

[31] Gundlach AL, Burazin TC, Jenkins TA, Berkovic SF (1995) Spatiotemporal alterations of central alpha 1-adrenergic receptor binding sites following amygdaloid kindling seizures in the rat: autoradiographic studies using [3H]prazosin. Brain Res 672: 214–227. 7749743 10.1016/0006-8993(94)01338-i

[32] Blendy JA, Grimm LJ, Perry DC, West-Johnsrud L, Kellar KJ (1990) Electroconvulsive shock differentially increases binding to alpha-1 adrenergic receptor subtypes in discrete regions of rat brain. J Neurosci 10: 2580–2586. 1974919 10.1523/JNEUROSCI.10-08-02580.1990 PMC6570269

[33] Rutecki PA (1995) Noradrenergic modulation of epileptiform activity in the hippocampus. Epilepsy Res 20: 125–136. 7750509 10.1016/0920-1211(94)00078-b

[34] Lerner JT, Sankar R, Mazarati AM (2008) Galanin and epilepsy. Cell Mol Life Sci 65: 1864–1871. 18500639 10.1007/s00018-008-8161-8 PMC11131733

[35] Sadegh M, Mirnajafi-Zadeh J, Javan M, Fathollahi Y, Mohammad-Zadeh M, et al (2007) The role of galanin receptors in anticonvulsant effects of low-frequency stimulation in perforant path-kindled rats. Neuroscience 150: 396–403. 17993248 10.1016/j.neuroscience.2007.09.068

[36] Mathie A (2007) Neuronal two-pore-domain potassium channels and their regulation by G protein-coupled receptors. J Physiol 578: 377–385. 17068099 10.1113/jphysiol.2006.121582 PMC2075148

[37] Churn SB, Sombati S, Jakoi ER, Severt L, DeLorenzo RJ (2000) Inhibition of calcium/calmodulin kinase II alpha subunit expression results in epileptiform activity in cultured hippocampal neurons. Proc Natl Acad Sci U S A 97: 5604–5609. 10779547 10.1073/pnas.080071697 PMC25875

[38] Carter DS, Haider SN, Blair RE, Deshpande LS, Sombati S, et al (2006) Altered calcium/calmodulin kinase II activity changes calcium homeostasis that underlies epileptiform activity in hippocampal neurons in culture. J Pharmacol Exp Ther 319: 1021–1031. 16971505 10.1124/jpet.106.110403

[39] del Castillo I, Cohen-Salmon M, Blanchard S, Lutfalla G, Petit C (1992) Structure of the X-linked Kallmann syndrome gene and its homologous pseudogene on the Y chromosome. Nat Genet 2: 305–310. 1303284 10.1038/ng1292-305

[40] Hu Y, Guimond SE, Travers P, Cadman S, Hohenester E, et al (2009) Novel mechanisms of fibroblast growth factor receptor 1 regulation by extracellular matrix protein anosmin-1. J Biol Chem 284: 29905–29920. 19696444 10.1074/jbc.M109.049155 PMC2785620

[41] Jian B, Nagineni CN, Meleth S, Grizzle W, Bland K, et al (2009) Anosmin-1 involved in neuronal cell migration is hypoxia inducible and cancer regulated. Cell Cycle 8: 3770–3776. 19844165 10.4161/cc.8.22.10066 PMC11998029

[42] Zucchini S, Buzzi A, Barbieri M, Rodi D, Paradiso B, et al (2008) Fgf-2 overexpression increases excitability and seizure susceptibility but decreases seizure-induced cell loss. J Neurosci 28: 13112–13124. 19052202 10.1523/JNEUROSCI.1472-08.2008 PMC3844742

[43] Matveeva EA, Vanaman TC, Whiteheart SW, Slevin JT (2007) Asymmetric accumulation of hippocampal 7S SNARE complexes occurs regardless of kindling paradigm. Epilepsy Res 73: 266–274. 17174072 10.1016/j.eplepsyres.2006.11.003 PMC1868484

[44] Whiteheart SW, Schraw T, Matveeva EA (2001) N-ethylmaleimide sensitive factor (NSF) structure and function. Int Rev Cytol 207: 71–112. 11352269 10.1016/s0074-7696(01)07003-6

[45] Aruga J, Mikoshiba K (2003) Identification and characterization of Slitrk, a novel neuronal transmembrane protein family controlling neurite outgrowth. Mol Cell Neurosci 24: 117–129. 14550773 10.1016/s1044-7431(03)00129-5

[46] Gall CM, Lynch G (2004) Integrins, synaptic plasticity and epileptogenesis. Adv Exp Med Biol 548: 12–33. 15250583 10.1007/978-1-4757-6376-8_2

[47] Kohling R, Nischt R, Vasudevan A, Ho M, Weiergraber M, et al (2006) Nidogen and nidogen-associated basement membrane proteins and neuronal plasticity. Neurodegener Dis 3: 56–61. 16909038 10.1159/000092094

[48] Librizzi L, Regondi MC, Pastori C, Frigerio S, Frassoni C, et al (2007) Expression of adhesion factors induced by epileptiform activity in the endothelium of the isolated guinea pig brain in vitro. Epilepsia 48: 743–751. 17386052 10.1111/j.1528-1167.2007.01047.x

[49] Dietrich JB (2002) The adhesion molecule ICAM-1 and its regulation in relation with the blood-brain barrier. J Neuroimmunol 128: 58–68. 12098511 10.1016/s0165-5728(02)00114-5

[50] Huh GS, Boulanger LM, Du H, Riquelme PA, Brotz TM, et al (2000) Functional requirement for class I MHC in CNS development and plasticity. Science 290: 2155–2159. 11118151 10.1126/science.290.5499.2155 PMC2175035

[51] Stevens B, Allen NJ, Vazquez LE, Howell GR, Christopherson KS, et al (2007) The classical complement cascade mediates CNS synapse elimination. Cell 131: 1164–1178. 18083105 10.1016/j.cell.2007.10.036

[52] Chu Y, Jin X, Parada I, Pesic A, Stevens B, et al (2010) Enhanced synaptic connectivity and epilepsy in C1q knockout mice. Proc Natl Acad Sci U S A 107: 7975–7980. 20375278 10.1073/pnas.0913449107 PMC2867906

[53] Salin P, Tseng GF, Hoffman S, Parada I, Prince DA (1995) Axonal sprouting in layer V pyramidal neurons of chronically injured cerebral cortex. J Neurosci 15: 8234–8245. 8613757 10.1523/JNEUROSCI.15-12-08234.1995 PMC6577943

[54] Prince DA, Tseng GF (1993) Epileptogenesis in chronically injured cortex: in vitro studies. J Neurophysiol 69: 1276–1291. 8492163 10.1152/jn.1993.69.4.1276

[55] Dityatev A (2010) Remodeling of extracellular matrix and epileptogenesis. Epilepsia 51 (Suppl 3)61–65. 20618403 10.1111/j.1528-1167.2010.02612.x

[56] Dityatev A, Frischknecht R, Seidenbecher CI (2006) Extracellular matrix and synaptic functions. Results Probl Cell Differ 43: 69–97. 17068968 10.1007/400_025

[57] Heck N, Garwood J, Loeffler JP, Larmet Y, Faissner A (2004) Differential upregulation of extracellular matrix molecules associated with the appearance of granule cell dispersion and mossy fiber sprouting during epileptogenesis in a murine model of temporal lobe epilepsy. Neuroscience 129: 309–324. 15501589 10.1016/j.neuroscience.2004.06.078

[58] Elmer E, Kokaia Z, Kokaia M, Lindvall O, McIntyre DC (1997) Mossy fibre sprouting: evidence against a facilitatory role in epileptogenesis. Neuroreport 8: 1193–1196. 9175112 10.1097/00001756-199703240-00027

[59] Veznedaroglu E, Van Bockstaele EJ, O’Connor MJ (2002) Extravascular collagen in the human epileptic brain: a potential substrate for aberrant cell migration in cases of temporal lobe epilepsy. J Neurosurg 97: 1125–1130. 12450035 10.3171/jns.2002.97.5.1125

[60] Tan Y, Shi L, Hussain SM, Xu J, Tong W, et al (2006) Integrating time-course microarray gene expression profiles with cytotoxicity for identification of biomarkers in primary rat hepatocytes exposed to cadmium. Bioinformatics 22: 77–87. 16249259 10.1093/bioinformatics/bti737

[61] Tan Y, Shi L, Tong W, Wang C (2005) Multi-class cancer classification by total principal component regression (TPCR) using microarray gene expression data. Nucleic Acids Res 33: 56–65. 15640445 10.1093/nar/gki144 PMC546133

[62] Peterson LE (2003) Partitioning large-sample microarray-based gene expression profiles using principal components analysis. Comput Methods Programs Biomed 70: 107–119. 12507787 10.1016/s0169-2607(02)00009-3

[63] Raychaudhuri S, Stuart JM, Altman RB (2000) Principal components analysis to summarize microarray experiments: application to sporulation time series. Pac Symp Biocomput: 455–466. 10.1142/9789814447331_0043 PMC2669932 10902193

[64] Huang DW, Sherman BT, Lempicki RA (2009) Systematic and integrative analysis of large gene lists using DAVID bioinformatics resources. Nat Protoc 4: 44–57. 19131956 10.1038/nprot.2008.211

[65] Dennis G Jr, Sherman BT, Hosack DA, Yang J, Gao W, et al (2003) DAVID: Database for Annotation, Visualization, and Integrated Discovery. Genome Biol 4: P3. 12734009 PMC193660

[66] Coudry RA, Meireles SI, Stoyanova R, Cooper HS, Carpino A, et al (2007) Successful application of microarray technology to microdissected formalin-fixed, paraffin-embedded tissue. J Mol Diagn 9: 70–79. 17251338 10.2353/jmoldx.2007.060004 PMC1867423

[67] Chung JY, Braunschweig T, Hewitt SM (2006) Optimization of recovery of RNA from formalin-fixed, paraffin-embedded tissue. Diagn Mol Pathol 15: 229–236. 17122651 10.1097/01.pdm.0000213468.91139.2d

[68] Xu J, Deng X, Demetriou AA, Farkas DL, Hui T, et al (2008) Factors released from cholestatic rat livers possibly involved in inducing bone marrow hepatic stem cell priming. Stem Cells Dev 17: 143–155. 18225978 10.1089/scd.2007.0094

